# The role of D3-type cyclins is related to cytokinin and the bHLH transcription factor SPATULA in *Arabidopsis* gynoecium development

**DOI:** 10.1007/s00425-024-04481-4

**Published:** 2024-07-09

**Authors:** Vincent E. Cerbantez-Bueno, Joanna Serwatowska, Carolina Rodríguez-Ramos, J. Erik Cruz-Valderrama, Stefan de Folter

**Affiliations:** 1 Unidad de Genómica Avanzada (UGA-Langebio), Centro de Investigación y de Estudios Avanzados del Instituto Politécnico Nacional, 36824 Irapuato, México; 2 Present Address: Department of Botany and Plant Sciences, University of California Riverside, Riverside, CA 92521 USA; 3 Present Address: Departamento de Ingeniería Genética, Unidad Irapuato, Centro de Investigación y de Estudios Avanzados del Instituto Politécnico Nacional, 36824 Irapuato, México; 4 Present Address: Departamento de Biología Molecular de Plantas, Instituto de Biotecnología, Universidad Nacional Autónoma de México, Avenida Universidad 2001, Colonia Chamilpa, 62210 Cuernavaca, Morelos México

**Keywords:** Carpel margin meristem (CMM), Cell cycle, Cell division, CYCD3, Cyclins, Cytokinin, Differentiation, Gynoecium, SPATULA

## Abstract

**Main conclusion:**

We studied the D3-type cyclin function during gynoecium development in *Arabidopsis* and how they are related to the hormone cytokinin and the transcription factor SPATULA.

**Abstract:**

Growth throughout the life of plants is sustained by cell division and differentiation processes in meristematic tissues. In *Arabidopsis*, gynoecium development implies a multiphasic process where the tissues required for pollination, fertilization, and seed development form. The Carpel Margin Meristem (CMM) is a mass of undifferentiated cells that gives rise to the gynoecium internal tissues, such as septum, ovules, placenta, funiculus, transmitting tract, style, and stigma. Different genetic and hormonal factors, including cytokinin, control the CMM function. Cytokinin regulates the cell cycle transitions through the activation of cell cycle regulators as cyclin genes. D3-type cyclins are expressed in proliferative tissues, favoring the mitotic cell cycle over the endoreduplication. Though the role of cytokinin in CMM and gynoecium development is highly studied, its specific role in regulating the cell cycle in this tissue remains unclear. Additionally, despite extensive research on the relationship between *CYCD3* genes and cytokinin, the regulatory mechanism that connects them remains elusive. Here, we found that D3-type cyclins are expressed in proliferative medial and lateral tissues. Conversely, the depletion of the three *CYCD3* genes showed that they are not essential for gynoecium development. However, the addition of exogenous cytokinin showed that they could control the division/differentiation balance in gynoecium internal tissues and outgrowths. Finally, we found that SPATULA can be a mechanistic link between cytokinin and the D3-type cyclins. The data suggest that the role of D3-type cyclins in gynoecium development is related to the cytokinin response, and they might be activated by the transcription factor SPATULA.

**Supplementary Information:**

The online version contains supplementary material available at 10.1007/s00425-024-04481-4.

## Introduction

The development of plant organs relies on cell division and differentiation in meristematic tissues (Gaillochet and Lohmann 2015). In *Arabidopsis*, the development of the gynoecium starts at the center of the floral meristem with the establishment of the gynoecium primordium at stage 6 of floral development (Smyth et al. 1990; Roeder and Yanofsky 2006; Alvarez-Buylla et al. 2010; Denay et al. 2017; Herrera-Ubaldo and de Folter 2022). By stage 7, the gynoecium primordium undergoes growth and division, giving rise to two distinct domains, referred to as medial and lateral domain (Bowman et al. 1999; Reyes-Olalde et al. 2013; Zúñiga-Mayo et al. 2019; Herrera-Ubaldo and de Folter 2022). These medial ridges subsequently merge to form a meristematic tissue known as the Carpel Margin Meristem (CMM). In the subsequent stages of development, CMM plays a pivotal role in generating various components, including the placenta, ovules, septum, transmitting tract, style, and stigma (Alvarez and Smyth 2002; Reyes-Olalde et al. 2013; Reyes-Olalde and de Folter 2019). The meristematic activity within the CMM has been characterized by the expression of specific genes and hormone activity, which regulate cell division and differentiation, akin to their functions in other meristematic tissues (Reyes-Olalde et al. 2013; Reyes-Olalde and de Folter 2019; Herrera-Ubaldo and de Folter 2022). In contrast to the medial region, the lateral region consists of 5–6 cell layers that will generate the valves (Bowman et al. 1999). The correct patterning and growth in the gynoecium axes are controlled by different genetic and hormonal factors (Moubayidin and Østergaard 2017; Reyes-Olalde et al. 2017, 2019; Herrera-Ubaldo and de Folter 2022).

Cytokinin, a hormone derived from adenine, plays diverse roles in plant development (El-Showk et al. 2013; Mok and Mok 2001; Kieber and Schaller 2018; Márquez-López et al. 2019; Wybouw and De Rybel 2019). Within the context of the gynoecium, cytokinin has been documented as a key regulator of gynoecium initiation and patterning (Marsch-Martinez et al. 2012; Zuñiga-Mayo et al. 2014, 2018; Marsch-Martínez and de Folter 2016; Müller et al. 2017; Reyes-Olalde et al. 2017; Gomez-Felipe et al. 2021; Carabelli et al. 2021; Herrera-Ubaldo and de Folter 2022). Moreover, cytokinin has been directly associated with genes involved in the development of various gynoecium tissues, including the CMM (Bartrina et al. 2011; Durán-Medina et al. 2017; Reyes-Olalde et al. 2017; Cuccinotta et al. 2020; Di Marzo et al. 2020; Cerbantez-Bueno et al. 2020; Herrera-Ubaldo and de Folter 2022). For instance, the transcription factor SPATULA (SPT) has been shown to function as a positive regulator of stem cell proliferation through the stimulation of cytokinin signaling (Schuster et al. 2015; Gaillochet et al. 2017). In the context of the CMM, SPT plays a critical role in facilitating cytokinin signaling and fostering the development of associated structures, such as the septum and transmitting tract (Heisler et al. 2001; Alvarez and Smyth 2002; Reyes-Olalde et al. 2017).

The role of cytokinin in meristematic tissues has also been related to the regulation of the cell cycle, promoting cell division in some tissues (shoot) and differentiation in others (root) (Schaller et al. 2014). The cell cycle in plants is a highly conserved and regulated four-step process that briefly consists of the duplication of the genome (S phase) and the production of two daughter cells (M phase), separated by G1 and G2 phases, respectively (Harashima et al. 2013; Gutierrez 2016; Sablowski and Gutierrez 2022). These transitions of the cell cycle in plants, as in other eukaryotes, are coordinated by the cyclin-dependent kinase proteins (CDKs), and depend on cyclin subunits, which change at different stages of the cell cycle (Sablowski and Gutierrez 2022). It has been found that cytokinin regulates both G1/S and G2/M transitions (Schaller et al. 2014). The regulation of the G1/S transition occurs through the induction of D3-type cyclins (Riou-Khamlichi et al. 1999; Scofield et al. 2013; Schaller et al. 2014). However, despite substantial evidence supporting the role of cytokinin in regulating the G2/M transition, the exact mechanism remains unclear (Hare and van Staden 1997; Laureys et al. 1998; Lipavská et al. 2011; Francis 2011; Schaller et al. 2014). An increase in DNA content can occur through multiple rounds of duplication without intervening mitosis, a phenomenon known as endoreduplication (Breuer et al. 2010, 2014; Edgar et al. 2014). This process typically leads to an increase in cell size and eventual differentiation into specific cell types (Sugimoto-Shirasu and Roberts 2003; Dewitte et al. 2007; Lang and Schnittger 2020). Therefore, the role of cytokinin in controlling G1/S and G2/M transitions is crucial in determining the commitment to mitosis or endoreduplication processes. This differential regulation can influence whether cell division or differentiation predominates in different organs (Schaller et al. 2014).

Some functions of D-type cyclins in plants have already been studied (Dewitte and Murray 2003; Dewitte et al. 2007). Among these, D3-type cyclins regulate the G1/S transition in the cell cycle by forming associations with CDK proteins to phosphorylate the retinoblastoma-related protein (RBR). This phosphorylation relieves the inhibition of the E2F transcription factor, thereby promoting entry into the S-cell cycle phase (de Jager et al. 2005; Schaller et al. 2014; Gutierrez and Sablowski 2022). In *Arabidopsis*, the D3-type cyclin group consists of three members: *CYCD3;1*, *CYCD3;2*, and *CYCD3;3* (Vandepoele et al. 2002). Their expression has been found in proliferative tissues and they appear to control the type of cell cycle, favoring the mitotic over the endoreduplication (Dewitte and Murray 2003; Dewitte et al. 2007; Scofield et al. 2013). The expression of D3-type cyclins is induced by endogenous signals, including plant hormones such as cytokinin (Riou-Khamlichi et al. 1999; Meijer and Murray 2000; Dewitte et al. 2007). Their depletion diminishes the ability of cytokinin to guide shoot formation. However, the overexpression of *CYCD3;1* prompts shoot formation from calli independently of cytokinin, suggesting an autonomy from cytokinin signaling (Riou-Khamlichi et al. 1999). Although the role of *CYCD3* genes in cell cycle progression has been reported as dispensable (Dewitte et al. 2007), they influence cell number, contribute to alternative cellular production and expansion processes, and mediate the effects of cytokinin. Despite extensive research on the relationship between *CYCD3* genes and cytokinin, the regulatory mechanism that connects them remains elusive. Furthermore, the specific function of *CYCD3* genes in the cell cycle within the context of gynoecium tissue development remains unexplored. This study focuses on elucidating the role of *CYCD3* genes and their relationship with cytokinin in gynoecium development while attempting to unveil the mechanistic link that bridges cytokinin and D3-type cyclins. We found that the transcription factor SPATULA (SPT) in part is involved.

## Materials and methods

### Plant lines and growth conditions

All reporter lines (*CYCD3;1::GUS*, *CYCD3;2::GUS*, and *CYCD3;3::GUS*) and mutants (*cycd3;1*, *cycd3;2*, *cycd3;3* single mutants, and *cycd3;1–3* triple mutant) were obtained from James Murray and have been described in Dewitte et al. (2007). The *spt-12* and *35S::SPT* seeds were provided by Karen Hallyday. The lines are in the Col-0 background, except *CYCD3;1::GUS*, which is in the L*er* background. All *Arabidopsis* and *Nicotiana* plants were grown in soil at ~ 23 °C under long-day conditions (16 h light/8 h dark) in a greenhouse or a growth chamber.

### GUS analysis

Gynoecia of different developmental stages were dissected and pre-fixed with cold acetone for 20 min, then rinsed and transferred into GUS substrate solution: 50 mM sodium phosphate pH 7, 5 mM, K3/K4 FeCN, 0.1% (v/v) Triton X-100, and 2 mM X-Gluc (Gold Biotechnology Inc.). After application of vacuum for 20 min, the samples were incubated at 37 °C for different times: ~ 1 week for *CYCD3;1::GUS*, ~ 22 h for *CYCD3;2::GUS*, and ~ 8 h for *CYCD3;3::GUS*.

### Histological analysis

Tissues were fixed in FAE (3.7% formaldehyde, 5% glacial acetic acid, and 50% ethanol, by vol.) with a vacuum for 15 min at 4 °C and then incubated for 60 min at room temperature. The material was rinsed with 70% ethanol and incubated overnight at 4 °C, followed by dehydration in a series of alcohol solutions (70, 85, 95, and 100% ethanol) for 60 min each, and embedded in Technovit as previously described (Marsch-Martínez et al. 2014). Then, 8 µm tissue sections were cut using low profile blades (Leica) on a Leica RM2035 microtome (Leica). Pictures were taken using a Leica DM600B microscope (Leica) with a DFC420C camera (Leica).

### Tissue staining

Tissues were treated as described in histological analysis. Then, transmitting tract staining was performed as previously described (Zúñiga-Mayo et al. 2012). In summary, tissue sections were stained with a solution of 0.5% Alcian blue (pH 3.1; Sigma-Aldrich) for 25 min and counterstained with a solution of 0.5% neutral red (Sigma-Aldrich) for 5 min. Slides were rinsed in water, air dried, mounted, and observed in a Leica DM600B microscope. Photos were taken with a DFC420C camera (Leica).

### Cytokinin treatment

Seeds were germinated and grown in soil under greenhouse conditions. One week after bolting, drops of BAP or mock solution were placed on the inflorescences once a day for 10 days for phenotype analyses. In the case of gene expression analysis (RT-qPCR), drops were placed only once, and the tissue was collected after 2 h. BAP solution contained 100 µM 6-benzylaminopurine; 0.01% Silwet L-77 (Lehle Seeds), and the mock solution contained only 0.01% Silwet L-77 in distilled water. The treated and control plants were grown under the same conditions.

### Gene expression analysis

For RT-qPCR analysis, floral buds in different stages were collected and total RNA was extracted using TRIzol (Invitrogen). The RNA was analyzed using an Open qPCR system (Chai Inc., Santa Clara, CA, USA) with qPCRBIO SyGreen 1-Step Go Hi-ROX (PCR Biosystems, Wayne, PA, USA) according to the manufacturer´s instructions. Three biological replicates and two technical replicates were done for each assay. Two or three replicates were analyzed using the 2^−ΔΔCT^ method. Target gene expression levels were normalized to ACTIN2/7. Statistical analyses were made using *t* tests or one-way ANOVA and Tukey’s or Dunnet’s test as a post hoc for multiple comparisons. Data were analyzed and plotted using Prism (GraphPad). Primer sequences are listed in Table S1.

### Phenotype analyses

Gynoecia and fruits from different plants of the wild type and mutant *cycd3;1–3* lines were collected after the 10 days of 6-benzyl amino purine (BAP) treatment. The gynoecia and fruits were evaluated in different phenotypical aspects: crests growth (ectopic tissue proliferation), apical–basal patterning, septum, transmitting tract, and ovule–funiculus phenotype. The identified effects were scored as the percentage of structures presenting mid-to-severe effects or slight-to-no effects in the analyzed tissues. Data analysis and plots were made using Prism (GraphPad).

### Y1H

Two different fragments of the *CYCD3;3* promoter (*CYCD3;3 I*, 1,540 bp, and *CYCD3;3 II*, 1,629 bp; see Table S1 for oligo sequences) were cloned in the pENTR/D-TOPO vector (Invitrogen), verified by sequencing, and introduced into the CZN1018 vector (pAbAi + Gateway site; Danisman et al. 2012) by Gateway LR recombination. The *SPT* cDNA fused to the Gal4 activation domain in pDEST22, was previously described (Herrera-Ubaldo et al. 2023). Yeast transformations were performed as previously described (de Folter and Immink 2011) using the strains PJ69A and PJ69α for SPT, and *CYCD3;3* promoters, respectively. Transformants were selected on SD-Gluc medium without tryptophan or uracil (SD-TRP or SD-URA). Mating was performed by droplets of each transformed yeast on YPAD medium, and the diploid selection was made on SD-TRP-URA plates. The final assay was done on SD-TRP-URA complemented with 150 ng/mL of Aureobasidin A (AbA; concentration obtained by a previous autoactivation assay). Yeast was grown at different dilutions at 30 °C, and after 5 days, interactions were scored. Haploid yeasts containing *CYCD3;3* fragments were grown in medium with and without AbA as negative and positive controls.

### Luciferase assay

For the luciferase assay constructs, a modified version of the *pGreen II—0800—Luc* was created. A 50 bp 35S minimum promoter was created annealing two 50 bp oligos that generated NotI 5′ and BamHI 3′ sticky ends (see Table S1 for sequences). Then, this 35S minimum promoter was cloned into the *pGreen II—0800—Luc* NotI 5′ and BamHI 3′ sites, resulting in the *mini35SpGreenII—0800—Luc* vector (see Fig. S1 for map). Each of the previously generated *CYCD3;3* fragments in the pENTR/DTOPO (Invitrogen) vector were subcloned into the ApaI 5′ and EcoRv 3′ sites of the *mini35SpGreenII—0800—Luc* vector.

The transient Luciferase expression assays were performed by the transient transformation of *N. benthamiana* leaves by *Agrobacterium* infiltration, performed as previously described (Espley et al. 2009) with minor modifications. In summary, an overnight culture of *Agrobacterium* was used to prepare an infiltration solution with 10 mM MgCl_2_, 10 mM MES, and 150 µM acetosyringone (pH 5.6). Each Luciferase vector was co-infiltrated with the previously described *35S::SPT* vector (Reyes-Olalde et al. 2017) into young and healthy *N. benthamiana* leaves. After 3 days, small discs were cut out of the infiltrated leaves (3 discs of ~ 6 mm diameter per sample) and homogenized in a 1X PBS solution. The liquid part of every sample was then combined in equal parts with the 2X TMCA solution to a final concentration of 100 mM Tris HCl (pH 7.8), 5 mM MgCl_2_, 250 µM CoA, and 150 µg/mL Luciferin-K (Gold BioTechnology Inc., St. Louis, MO, USA) in a 96-well plate. The plate was read in a Luminometer LmaxII384 (Molecular Devices) with each experiment in triplicate and using spaces without samples as blank references. At least three leaves at the same developmental stage were used for each combination, and the experiments were repeated at least two times. The results were analyzed and plotted using Prisma (GraphPad).

## Results

### D3-type cyclin genes are differentially expressed during gynoecium development

**Fig. 1:**
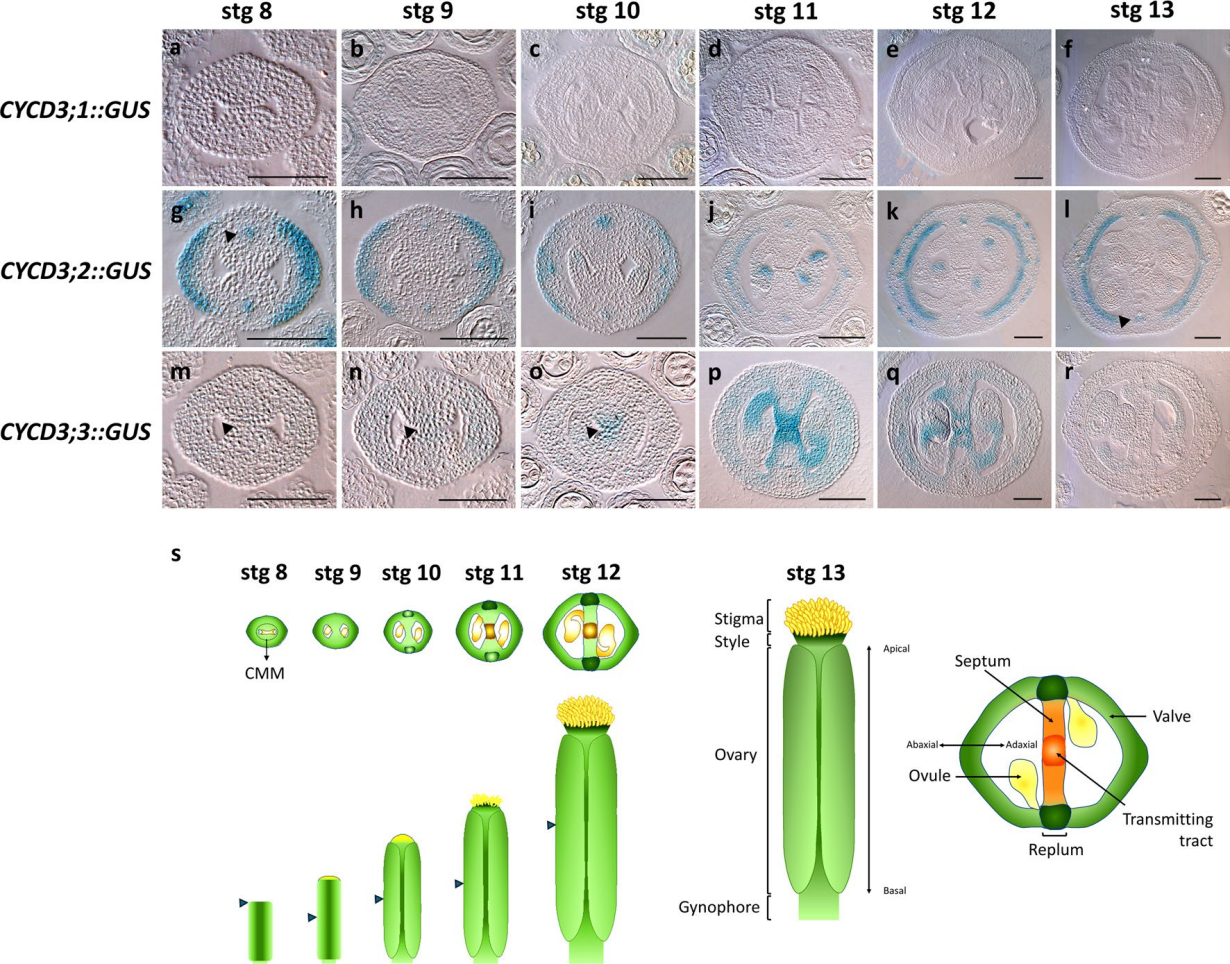
Expression profile of the D3-type cyclin genes during gynoecium development. **a**–**r** Transverse sections of the transcriptional fusion reporters *CYCD3;1::GUS* (**a**–**f**), *CYCD3;2::GUS* (**g**–**l**), and *CYCD3;3::GUS* (**m–r**) in stages 8 to 13 (left to right) of gynoecium development. Arrowheads represent vasculature in development (**g**–**l**) and medial tissue in development (**m**–**o**). Graphical representation of gynoecium development and its tissues (**s**). The position of the transverse cuts is represented by arrowheads in (**s**). Scale bars = 50 µm

The meristematic activity within the CMM has been inferred primarily from the expression of genes and hormone activity linked to cell division and differentiation (Reyes-Olalde et al. 2013; Reyes-Olalde and de Folter 2019; Herrera-Ubaldo and de Folter 2022). However, specific cell cycle-related markers have not been definitively identified in this tissue. In the context of *Arabidopsis*, the D-type cyclins have been recognized for their role in governing entry into the S phase (Schaller et al. 2014), and their expression appears to be associated with actively proliferating tissues (Dewitte and Murray 2003; Dewitte et al. 2007; Menges et al. 2006). Among this cyclin group, the D3-type cyclins have been observed to be expressed in reproductive tissues (Collins et al. 2012; Dewitte and Murray 2003), and data available in databases confirm the gene expression of D3-type cyclins at various gynoecium developmental stages (Fig. S2). Nevertheless, the precise localization of these cyclins within this tissue has not been extensively characterized. Here, we studied the expression of the three *Arabidopsis* D3-type cyclins during gynoecium development using transcriptional promoter GUS fusions (Dewitte et al. 2007). In general, while we obtained the expression patterns for *CYCD3;2* and *CYCD3;3* (Fig. 1), we could not detect any GUS signal in the *CYCD3;1::GUS* gynoecia analysed (Fig. 1a–f) due to the apparent low expression or absence of *CYCD3;1* in the analysed tissues (Dewitte et al. 2007; Fig. S2). To discard any putative silencing of the *CYCD3;1::GUS* line, we performed GUS staining on seedlings and found GUS expression in the leaf tips, as reported before (Fig. S2; Dewitte et al. 2007).

The signal of *CYCD3;2::GUS* was generally observed throughout gynoecium development, being detected in the vascular bundles, ovules, and valves (Fig. 1g–l). There were slight variations in expression within different tissues across various developmental stages. Notably, the expression in vascular bundles persisted from the early to mature stages of gynoecium development. However, the GUS activity in the valves and ovules exhibited stage-specific differences. At the early stage (stage 8), high GUS activity was observed in the abaxial area of the valves and the medial vasculature bundles (Fig. 1g). Subsequently, at stage 9, the activity in the valves and vascular bundles decreased, but it remained prominent in the abaxial area (Fig. 1h). By stage 10, the GUS activity further decreased in the valves, but increased in the vascular bundles, and became noticeable in developing ovules (Fig. 1i). *CYCD3;2* activity continued to be present in the valves at stage 11, encompassing both the inner and outer layers (Fig. 1j). This pattern persisted through stage 12, with a slight increase at this later stage (Fig. 1k). In stages 11 and 12, the activity of *CYCD3;2* was visible in ovules and vascular bundles too (Fig. 1j, k). At stage 13, the expression in the inner cells of the valves was maintained, but the activity decreased in the outer layer of cells; at this stage, the activity in ovules vanished (Fig. 1l).

The expression of *CYCD3;3* was observed in certain stages of gynoecium development. In general, its activity was concentrated in the tissues of the medial domain such as in the CMM, developing septum, funiculi, and the base of the ovule (Fig. 1m–r). Additionally, some activity was detected in the valves during specific developmental stages. At stage 9, perhaps even at stage 8, faint GUS signal was visible in the CMM (Fig. 1m, n). At stage 10, *CYCD3;3*::*GUS* activity was more clearly observed in the CMM/developing septa and in ovule primordia, with slight activity in the valves too (Fig. 1o). By stage 11, *CYCD3;3* expression intensified and remained prominent in the central regions of the medial tissues, in the funiculi, and certain layers of the septum. During this stage, there was also a mild presence of expression in the valves (Fig. 1p). In stage 12, *CYCD3;3* expression was prominently localized in the transmitting tract and funiculi, with a subtle presence in the septum and valves (Fig. 1q). At stage 13, the *CYCD3;3* expression was almost absent, only some faint expression in the funiculi (Fig. 1r).

In summary, distinct expression patterns were observed for each of the three D3-type cyclins, which generally align with information available in the database (Fig. S2). Specifically, *CYCD3;2* was predominantly expressed in lateral tissues, whereas *CYCD3;3* was more found in medial tissues. These different expression patterns suggest potential differences in the spatiotemporal functions of D3-type cyclins in the development of gynoecium tissues.

### D3-type cyclin genes are induced by cytokinin in the gynoecium

Cytokinin has been implicated in the regulation of the cell cycle, particularly in governing the G1/S and G2/M transitions (Schaller et al. 2014). Specifically, cytokinin-induced expression of the D3-type cyclin genes has been described in *Arabidopsis* seedlings as a mechanism controlling the G1/S transition (Riou-Khamlichi et al. 1999).

**Fig. 2:**
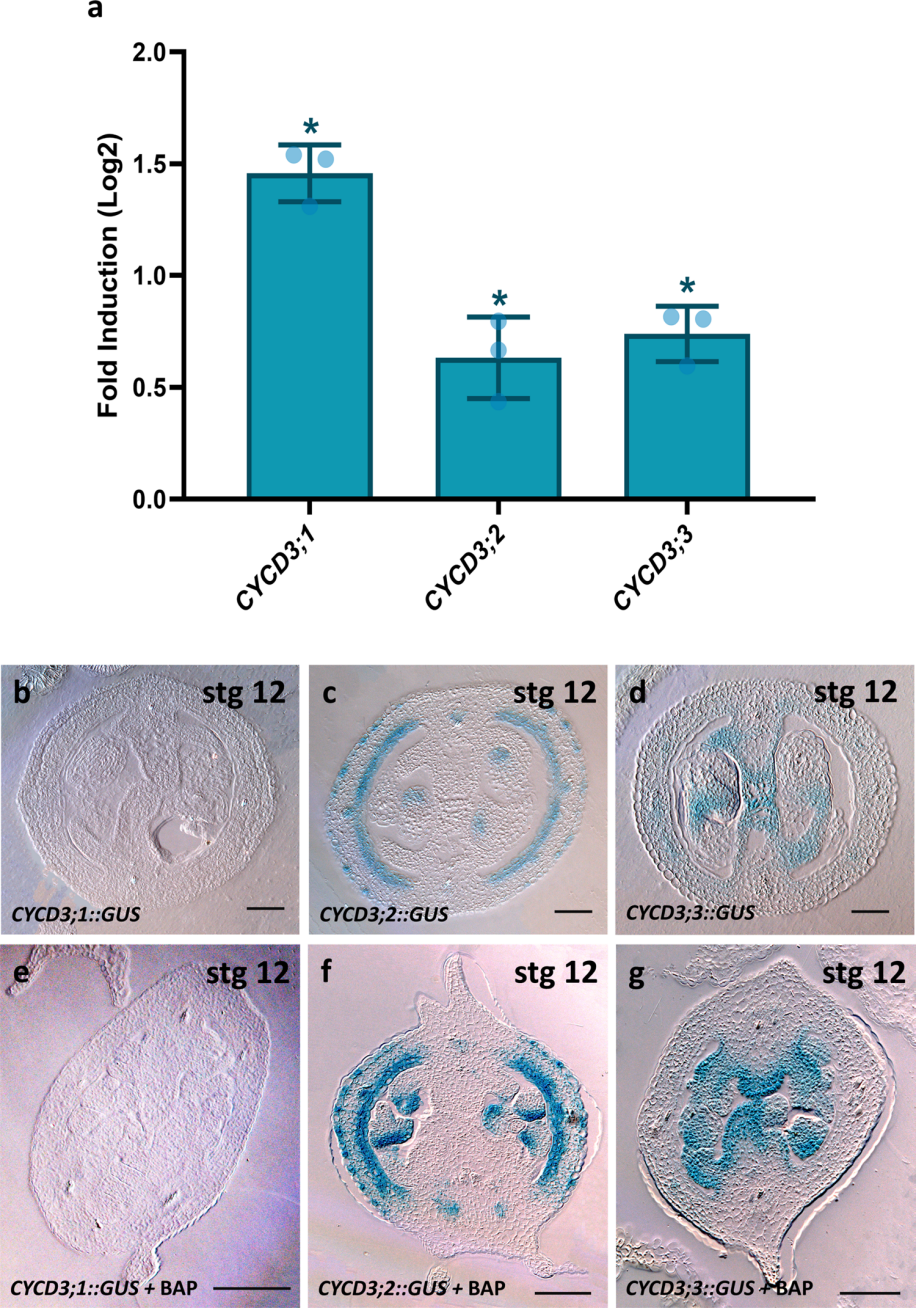
D3-type genes are positively regulated by exogenous cytokinins. **a** RT-qPCR fold change values for the three D3-type genes after 2 h of BAP treatment in inflorescences with respect to mock values. *CYCD3;1* (1.45 ± 0.12SD Log2FC, *n* = 3), *CYCD3;2* (0.63 ± 0.18SD Log2FC, *n* = 3), and *CYCD3;3* (0.73 ± 0.12SD Log2FC, *n* = 3). Significant differences were determined by a *t* test considering *P* < 0.05. **e**–**g** Transverse sections of the transcriptional fusion reporters after 10-day BAP treatment: *CYCD3;1::GUS* (**e**), *CYCD3;2::GUS* (**f**), and *CYCD3;3::GUS* (**g**). **b–d** Transverse sections of the *CYCD3* transcriptional fusion reporters without BAP treatment, corresponding with e, k, q in Fig. 1. The position of the transverse cuts for stage 12 is represented by arrowheads in Fig. 1s. Scale bars = 50 µm

To investigate whether D3-type cyclins are similarly regulated by cytokinin in the context of the gynoecium, we conducted experiments involving the application of BAP (6-benzyl amino purine) to various *Arabidopsis* lines. First, we isolated RNA from wild-type inflorescence tissue 2 h after BAP application and examined its impact on the expression of the three D3-type cyclins by RT-qPCR. Notably, we observed a significant increase in the gene expression of *CYCD3;1* (1.45 ± 0.12 Log2FC), *CYCD3;2* (0.63 ± 0.18 Log2FC), and *CYCD3;3* (0.73 ± 0.12 Log2FC), as illustrated in Fig. 2a. This suggests that the regulation of these three genes by cytokinin occurs in floral tissues and can be inferred to also take place in the gynoecium.

To further investigate this, we examined the *promoter::GUS* lines of the three D3-type cyclins after BAP application. As anticipated, following a 10-day BAP application, we observed the emergence of ectopic tissues originating from the replum, a phenomenon previously documented (Marsch-Martínez et al. 2012; Zúñiga-Mayo et al. 2014; Cerbantez-Bueno et al. 2020). Subsequent to the GUS staining and histological procedures, the *CYCD3;1*::*GUS* line maintained showing no signal at the gynoecium stage 12 or other stages analysed (Figs. 2b, e and S3). This outcome suggests that the cytokinin-mediated induction observed in the RT-qPCR analysis of *CYCD3;1* might be taking place in other tissues used for this experiment such as the meristem (Dewitte et al. 2007). In the case of *CYCD3;2* and *CYCD3;3*, we did observe an increased signal in gynoecia, in line with the obtained RT-qPCR results (Fig. 2c, d, f, g). Besides increased GUS signal intensity for *CYCD3;2* in the valves and vascular bundles at stage 12, we observed seemingly additional vascular bundles marked by *CYCD3;2* expression (Fig. 2f). This effect was also present in other developmental stages (Fig. S3). These additional vascular bundles in the repla region corresponded with the locations of the ectopic outgrowths, where *CYCD3;2* was similarly expressed in later fruit developmental stages (Fig. S4). The GUS signal of *CYCD3;3* was also increased after exogenous cytokinin treatment, maintaining the same pattern in the medial domain at stage 12 (Fig. 2g), and other stages analysed (Fig. S3). Nevertheless, the GUS signal was not observed in the ectopic outgrowths nor in the valves (Fig. S4).

### D3-type cyclins role in gynoecium development

The D-type cyclin group comprises ten genes classified into seven groups (Vandepoele et al. 2002). Their function has been associated with cell proliferation during the G1 phase (Meijer and Murray 2000). Among the D-type cyclins, the D1 and D3 types have been notably linked to reproductive development (Soni et al. 1995; Meijer and Murray 2000; Gaudin et al. 2000). Although D1-type cyclins were also observed to be expressed in floral tissues, though to a lesser extent (Fig. S2), here we focus on the role of D3-type cyclins in gynoecium development. To investigate this, we examined the phenotypes of the three *CYCD3* single mutants and the *cycd3;1–*3 triple mutant.

**Fig. 3:**
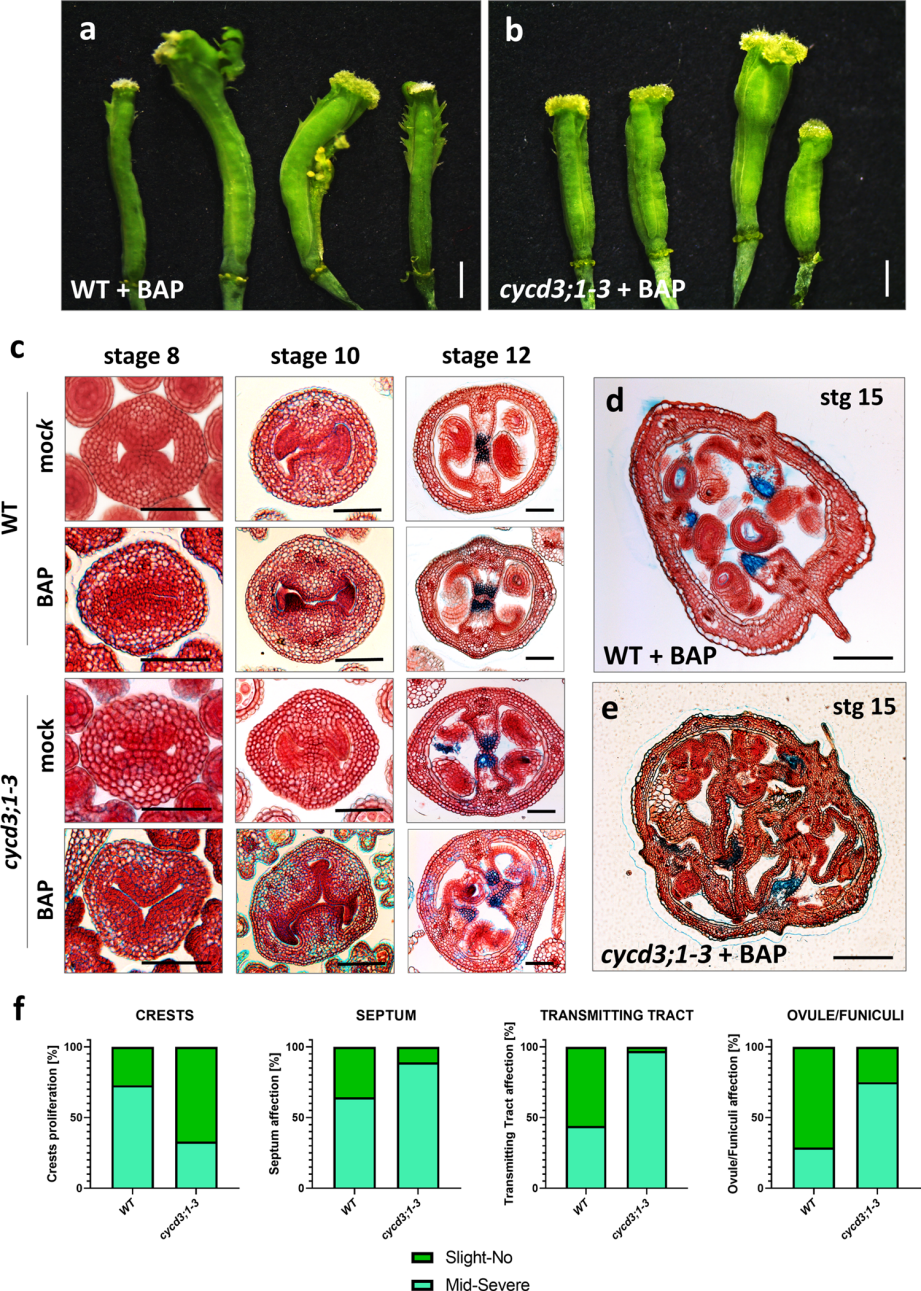
The role of the D3-type cyclins is related to cytokinin during gynoecium development. Phenotypes of gynoecia of WT (**a**) and *cycd3;1–3* triple mutant (**b**) after 10-day treatment of BAP. **c** Transverse sections of the WT (top) and *cycd3;1–3* triple mutant (bottom) during early (stage 8), mid (stage 10), and mature (stage 12) stages of gynoecium development after 10-day BAP or mock treatment. The position of the transverse cuts for each stage is represented by arrowheads in Fig. 1s. Transverse sections of fruits (stage 15) of WT (**d**) and *cycd3;1–3* triple mutant (**e**) after 10-day BAP treatment. **f** Percentage of different phenotypes found in the WT and *cycd3;1–3* triple mutant after 10-day BAP treatment. Phenotypes were classified artificially in two groups: slight-to-no phenotype (Slight-No), and mid-to-severe phenotype (mid–severe). *n* = 60 for all lines. Scale bars = 1 mm (**a**, **b**), 50 µm (**c**–**e**)

The *cycd3;1, cycd3;2,* and *cycd3;3* single and *cycd3;1–3* triple mutants have been previously described and recognized as null alleles (Dewitte et al. 2007). These mutants did not exhibit clear differences in inflorescence, flower, fruit, or external and internal gynoecium morphology in comparison to the wild type (Figs. S5 and S6). However, concerning the appearance of the cells in the gynoecium in the triple *cycd3;1–3* mutant, they appear to be bigger, and less in number, at early stages of gynoecium development; clearly visible in the medial and lateral domains at stage 8 gynoecium (Figs. 3c and S5). This phenotype of bigger cells without affecting organ size has already been described for other organs in this triple mutant (Dewitte et al. 2007) and supports the idea of yet a specific role for the D3-type cyclins in the gynoecium of *Arabidopsis*.

This observation suggests that the D3-type cyclins might have redundant or partial redundant roles during gynoecium development, and support the notion that these D3-type cyclins are not essential for the progression of the cell cycle, as previously proposed (Dewitte et al. 2007).

### D3-type cyclins role related to cytokinin during gynoecium development

To obtain more insight into the function of the D3-type cyclins in relation to cytokinin in the gynoecium, we examined the effects of applying exogenous cytokinin in the triple D3-type cyclin mutant.

Following a 10-day BAP application in wild-type and *cycd3;1–3* triple mutant plants, we observed a phenotype in the inflorescence that exhibit overgrown gynoecia (larger) both in wild-type and *cycd3;1–3* triple mutant background (Fig. S7). In addition, most of the wild-type gynoecia showed crest growth (Fig. 3a, f), or so-called ectopic proliferative tissue from the repla, as previously described (Marsch-Martínez et al. 2012). However, in the *cycd3;1–3* triple mutant, we observed no tissue proliferation in most of the gynoecia analysed (Fig. 3b, f). Subsequently, we made transverse sections of the treated gynoecia and fruits to gain deeper insights into the inner morphology. In general, the application of BAP induced the proliferation of ectopic tissues within the gynoecium, in the medial region and appeared to impact the balance between proliferation and differentiation in the *cycd3;1–3* triple mutant. The *cycd3;1–3* triple mutant gynoecia exhibited affected septum development, resulting in fruits with greatly altered septum structures (Fig. 3e). Though we did observe mild defects in septum development in gynoecia and fruit in the wild-type (e.g., wider septum, bigger replum and in some cases a third septum primordium formation, ~ 65%, *n* = 60, Fig. 3c, d), the effects in septum development were more severe and frequent in the *cycd3;1–3* triple mutant line (Fig. 3f, ~ 90%, *n* = 60). The impact on the transmitting tract led to a bifurcated structure in the gynoecium and a consequent aberrant arrangement of this tissue in the fruits of most of the *cycd3;1–3* samples analysed (Fig. 3c, f, 97%, *n* = 60), whereas an effect was observed in only some of the wild-type gynoecia and fruit samples (Fig. 3c, f, 43%, *n* = 60). The development of the ovule and funiculus was also affected; a shorter ovule-funiculus primordia in mid-stages of gynoecium development marked a delay in the development of these structures in both the wild-type and the *cycd3;1–3* mutant line after BAP application (Fig. 3c), which led to a malformation of ovules and funiculi in the fruits (Fig. 3d, e). Intriguingly, the *cycd3;1–3* mutant displayed this phenotype more frequently and severely (Fig. 3f, ~ 75%, *n* = 60) than the wild-type gynoecia and fruits analysed (Fig. 3f, ~ 30%, *n* = 60).

In summary, the *cycd3;1–3* triple mutant resulted hypersensitive to cytokinin application. This suggests that the D3-type cyclins have a correlation with the cytokinin response. This could be associated with the correct patterning of the gynoecium’s internal tissues, which is simultaneously regulated by cytokinin (Reyes-Olalde and Folter 2019). Other genes related to this cytokinin response process, such as *SPATULA* (*SPT*) and B-type *ARABIDOPSIS RESPONSE REGULATORS* (*ARRs*), have been reported to alter inner tissue patterning when not functional (Reyes-Olalde et al. 2017). This suggests that the D3-type cyclins could have a relationship with cytokinin, and some other genetic factors involved in the cytokinin response.

### The transcription factor SPATULA regulates D3-type cyclin gene expression

SPATULA (SPT) is a bHLH transcription factor known to play a role in various aspects of plant development (Groszmann et al. 2010). Within the gynoecium, *SPT* is expressed in the medial tissues (Heisler et al. 2001; Groszmann et al. 2010). The function of SPT has been directly associated with cytokinin, as it facilitates the hormonal response in CMM development by activating the B-type *ARR1* gene (Reyes-Olalde et al. 2017). The *spt* single mutant displays impairments in the development of medial tissues, such as the septum and transmitting tract (Alvarez and Smyth 1999; Heisler et al. 2001). In addition, the *spt* mutant, when treated with BAP, does not exhibit the characteristic cell proliferation originating from the replum, as is typically observed in the wild-type (Reyes-Olalde et al. 2017). A similar lack of response to BAP was observed in the *cycd3;1–3* triple mutant (Fig. 3). Furthermore, a portion of the expression of *SPT* overlaps with the expression of the *CYCD3;3* gene in the CMM and its derived tissues (Fig. 1). These areas are known to have cytokinin activity reported by a *TCS::GFP* line (Marsch-Martínez et al. 2012; Reyes-Olalde et al. 2017). In the following experiments, we studied a possible relationship between SPT and the *CYCD3* genes.

**Fig. 4:**
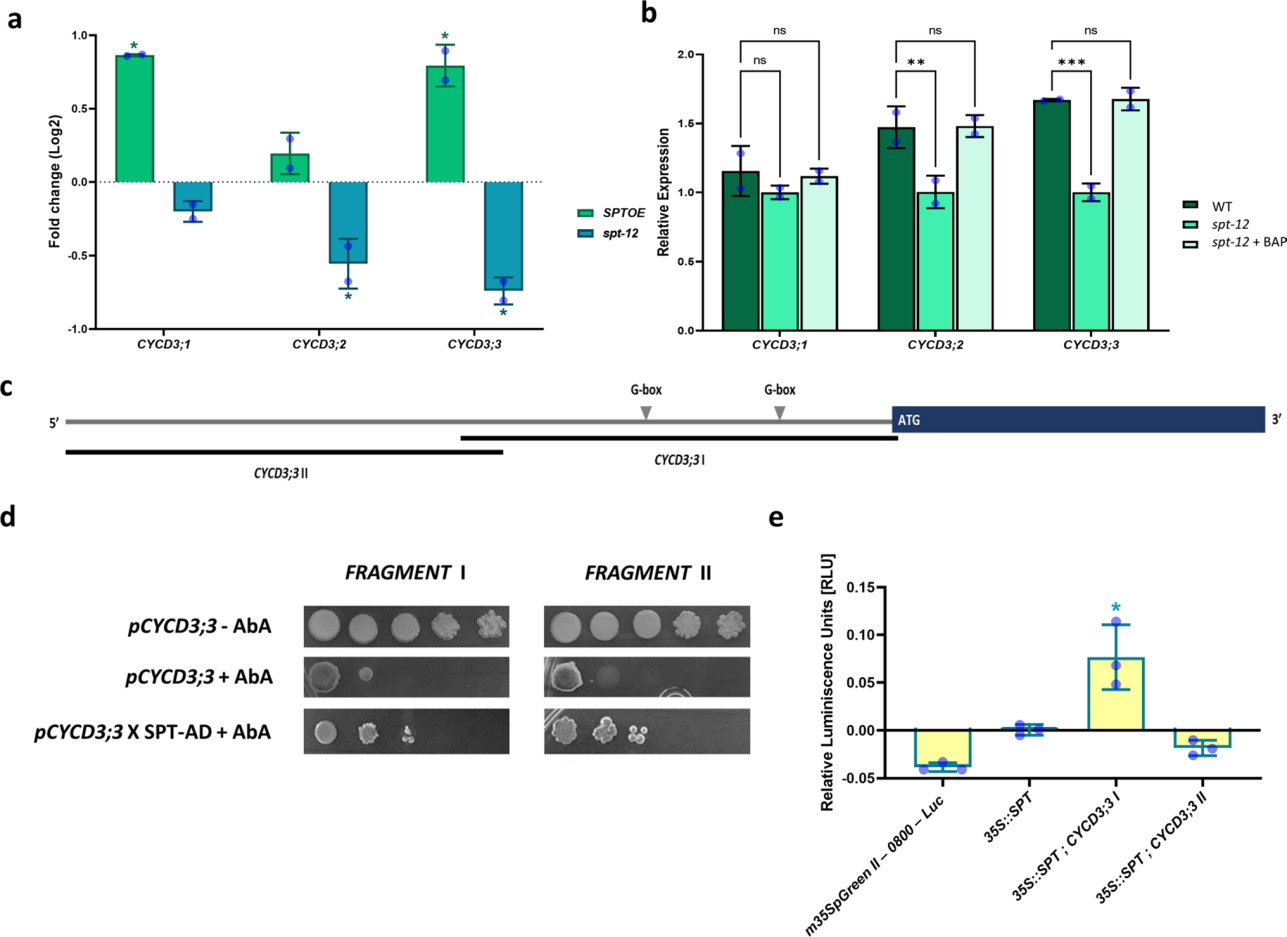
The transcription factor SPATULA regulates D3-type cyclin gene expression. **a** RT-qPCR fold change values for the three D3-type cyclins in SPATULA overexpression (SPTOE) and SPATULA mutant (*spt-12*) background with respect to the base values (WT values). SPTOE = *CYCD3;1* (0.86 ± 0.007SD Log2FC, *n* = 2), CYCD3;2 (0.19 ± 0.14SD Log2FC, *n* = 2), and *CYCD3;3* (0.79 ± 0.14 Log2FC, *n* = 2). *spt-12* = *CYCD3;1* (− 0.20 ± 0.07SD Log2FC, *n* = 2), *CYCD3;2* (− 0.55 ± 0.16SD Log2FC, *n* = 2), and *CYCD3;3* (− 0.74 ± 0.09SD Log2FC, *n* = 2). Significant differences were determined by a *t* test considering *P* < 0.05. **b** RT-qPCR relative expression values for the three D3-type cyclins in WT, *spt-12* and *spt-12* + 2 h BAP treatment background. *CYCD3;1* (WT = 1.16 ± 0.18SD; *spt-12* = 1.00 ± 0.04SD; *spt-12* + BAP = 1.11 ± 0.05SD; *n* = 2); *CYCD3;2* (WT = 1.47 ± 0.15SD; *spt-12* = 1.00 ± 0.11SD; *spt-12* + BAP = 1.48 ± 0.07SD; *n* = 2); *CYCD3;3* (WT = 1.67 ± 0.008SD; *spt-12* = 1.00 ± 0.06SD; *spt-12* + BAP = 1.67 ± 0.008SD; *n* = 2). Significant differences were determined by a two-way ANOVA followed by Dunnett’s multiple comparison analysis, considering *P* < 0.05 (** = 0.005; *** = 0.0005). **c** Graphical representation of the promoter portion considered for the *CYCD3;3* gene in the protein–DNA interaction analyses pointing out the location of the G-boxes and the regions amplified for *CYCD3;3 I* and *CYCD3;3 II* promoter segments. **d** Y1H assay represented by yeast growth in serial dilutions for the SPT and *CYCD3;3 I* and *CYCD3;3 II* interactions in positive control (*CYCD3*—AbA), negative control (*CYCD3* + AbA), and the interaction analyses (*CYCD3* × SPT + AbA). **e** Luciferase assay for SPT and the *CYCD3;3 I* and *CYCD3;3 II* interactions. Relative luminescence values for the empty vector (*mini35SpGreenII-0800-Luc*, − 0.038 ± 0.005SD, *n* = 3), *35S::SPT* vector by itself (*35S::SPT,* 0.001 ± 0.006SD, *n* = 3) and the interactions *35S::SPT* × *CYCD3;3 I* (0.077 ± 0.034SD, *n* = 3), and *35S::SPT* × *CYCD3;3 II* (− 0.018 ± 0.008SD, *n* = 3). Significant differences were determined by one-way ANOVA and Tukey as post hoc, considering *P* < 0.05. **a**, **b**, **e** Error bars represent SD

First, we performed expression analysis by RT-qPCR in perturbation experiments of SPT. For this, we extracted RNA from inflorescence tissue of *SPT* overexpression (*SPTOE*) and *spt-12* mutant lines, followed by RT-qPCR. Our findings showed that the overexpression of *SPT* triggered an increase in the expression of two out of the three D3-type cyclins: *CYCD3;1* (0.86 ± 0.007 Log2FC) and *CYCD3;3* (0.79 ± 0.14 Log2FC) (Fig. 4a). On the other hand, in the *spt-12* mutant, the expression of two out of three D3-type cyclins was reduced: *CYCD3;2* (− 0.55 ± 0.16 Log2FC) and *CYCD3;3* (− 0.74 ± 0.09 Log2FC), relative to wild-type (Fig. 4a). In essence, *SPT* positively regulated the expression of *CYCD3;1*, but this gene remained unaffected in the absence of *SPT*. We have mentioned that *CYCD3;1::GUS* was not found in the gynoecium, suggesting a regulatory role of SPT on this gene in meristematic tissues. Conversely, the scenario was different for *CYCD3;2* expression, where *SPT* overexpression had no impact on *CYCD3;2* expression, but the *spt-12* mutant led to a decrease. For *CYCD3;3*, this gene exhibited both positive and negative responses in the *SPTOE* and *spt-12* backgrounds, respectively.

All three D3-type cyclins were upregulated by cytokinin (Fig. 2a). To investigate whether SPT is essential for cytokinin-mediated regulation of the three different D3-type cyclins, we applied BAP to the *spt-12* mutant and conducted RT-qPCR. After 2 h of BAP application, the relative expression level of *CYCD3;1* remained unchanged, compared to the values observed in both *spt-12* and wild-type samples (Fig. 4b). In contrast, the expression levels of *CYCD3;2* and *CYCD3;3* in the *spt-12* + BAP sample exhibited alterations and reached levels similar to those in the wild-type (Fig. 4b). This implies that *CYCD3;1* relies on SPT for its regulation by cytokinin, but in the case of *CYCD3;2* and *CYCD3;3*, SPT function can potentially be replaced by other factors in their regulation following BAP application. Thus, it appears that SPT plays distinct regulatory roles for each of the D3-type cyclins.

**Fig. 5:**
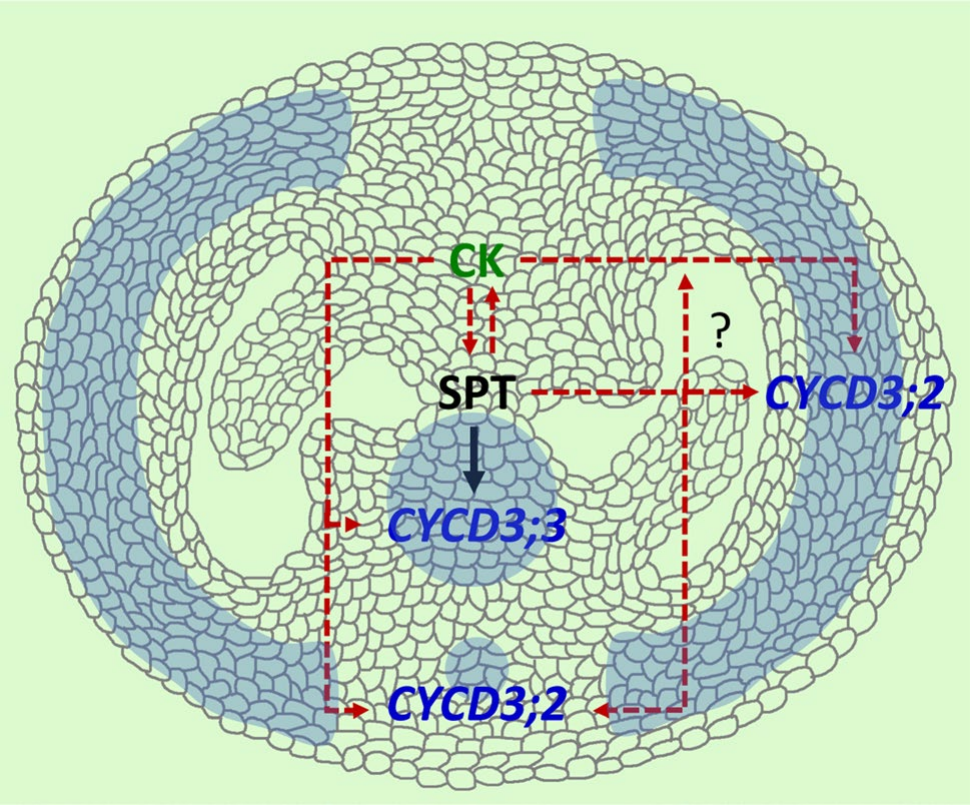
A model of the regulation of D3-type cyclins by cytokinin (CK) and the transcription factor SPATULA (SPT) in the gynoecium. The location of the gene or hormone represents the reported location. Dotted arrows represent indirect regulation and non-dotted arrows represent direct regulation. The light blue color indicates the expression of *CYCD3;2* or *CYCD3;3* based on transcriptional GUS fusion lines

Transcription factors often exert direct control over gene expression by binding to their promoters and thereby regulating transcription. Conventionally, SPT has been known to predominantly bind to specific motifs featuring a G-box sequence (CACGTG) (Girin et al. 2011; Reymond et al. 2012). We conducted an analysis of the upstream regions starting from the ATG of the *CYCD3;1*, *CYCD3;2*, and *CYCD3;3* genes. In the regulatory regions examined for the *CYCD3;1* and *CYCD3;2* genes, we did not identify any G-boxes. However, in the regulatory region of the *CYCD3;3* gene, we located two G-boxes at positions − 443 and − 912 bp (Fig. 4c). Despite the fact that SPT may not be indispensable for the regulation of *CYCD3;3* after cytokinin application, there exists a strong connection: they exhibit co-expression, and changes in *SPT* expression consistently influence *CYCD3;3*. To assess whether the regulation of *CYCD3;3* by SPT is a direct process, we carried out protein-DNA interaction assays. We amplified an approximately 1,500 bp fragment encompassing the SPT binding motifs of the *CYCD3;3* gene (*CYCD3;3 I*; Fig. 5c) and employed it to test the interaction via Y1H and Luciferase transactivation assays. We amplified another 1,500 bp fragment upstream of the other fragment from the same gene (*CYCD3;3 II*; Fig. 5c) as a control. In the Y1H assay, we observed yeast growth in both analysed fragments (Fig. 4d), indicating that SPT could bind to regions in both fragments and activate Aureobasidin A (AbA) resistance gene. In addition, in the Luciferase transactivation assay, we exclusively observed significant luminescence with the *CYCD3;3 I* fragment (Fig. 4e), signifying that SPT binds to this specific region where the binding sites are situated and activates gene transcription. These findings suggest that SPT directly regulates the expression of *CYCD3;3*.

In summary, we found a close association between the D3-type cyclins and the transcription factor SPT, with SPT able to directly regulate the expression of the *CYCD3;3* gene. Furthermore, it appears that cytokinin modulates D3-type cyclins through different mechanisms, depending on the presence or absence of SPT. This model of regulation is summarized in Fig. 5.

## Discussion

As for other organs, the formation and correct patterning of the gynoecium in *Arabidopsis* depends on the correct regulation of cell division and differentiation (Gaillochet and Lohmann 2015; Reyes-Olalde and de Folter 2019). Several factors responsible for governing the meristematic activity and ensuring the correct patterning of this tissue have already been identified (Reyes-Olalde and Folter 2019; Herrera-Ubaldo and de Folter 2022). This study focuses on investigating the role of D3-type cyclins and their connection to the hormone cytokinin and the transcription factor SPT in the development of the *Arabidopsis* gynoecium.

### *CYCD3* genes might play a role in the differentiation of medial and lateral tissues of the gynoecium

Previous analysis of the *cycd3;1–3* triple mutant allowed to determine a role of the *CYCD3* genes in cell proliferation, endoreduplication, and shoot apical meristem (SAM) maintenance (Dewitte et al. 2007). During gynoecium development, the cells of the CMM possess meristematic activity at early stages (Reyes-Olalde and de Folter 2019; Herrera-Ubaldo and de Folter 2022). Consequently, the development of inner tissues depends on a correct function and maintenance of the CMM. In our results we did not find apparent effects of the mutations of the three *CYCD3* genes in the inner tissues of the mature gynoecium (Figs. 3 and S5). However, at stage 8, a clear increase in cell size and reduction in cell number was observed, including in the CMM. Low activity of the *CYCD3;3* promoter was observed in the CMM, starting to be visible at stage 9 and 10, which is rather late when thinking in CMM formation. This suggests that the role of the D3-type cyclins in the gynoecium might have no relationship with CMM formation or maintenance, which partially explains the absence of affection in the CMM-derived tissues in the *cycd3;1–3* triple mutant. These data might suggest that *CYCD3* genes are not essential for the development of internal organs in the gynoecium. Additionally, the expression of *CYCD3;1* was not found in the gynoecium, though it has been previously reported to play essential roles in other tissues (Masubelele et al. 2005).

Furthermore, based on transcriptional promoter fusions, we found the expression of the *CYCD3* genes in other tissues. *CYCD3;2* gene was expressed in the lateral region and vasculature from early to mature stages of gynoecium development (Fig. 1g–l), and *CYCD3;3* was found in the CMM, septum, transmitting tract and ovules in development (Fig. 1m–r). As mentioned, we did not find any clear phenotype in the *cycd3;2* or *cycd3;3* single mutants, nor in the *cycd3;1–3* triple mutant in these tissues (possibly to redundancy with other *CYCD* genes expressed in these tissues, Fig. S2). However, the different expression patterns and the apparent increased cell size in *cycd3;1–3* led us to some speculations about their function in gynoecium development. The correct development of the gynoecium structures depends on an equilibrium between cell division and differentiation. In the cell cycle, *CYCD3* genes have been characterized to control the mitotic cycle duration and the transition to endocycles in different tissues (Schnittger et al. 2002; Dewitte et al. 2007), and endocycles are usually related with an increase in cell size and differentiation (Sugimoto-Shirasu and Roberts 2003; Dewitte et al. 2007; Lang and Schnittger 2020; Robinson et al. 2018). If the role of *CYCD3* genes is not proliferative, then it could be to regulate the transition from mitotic cycles to endocycles during the differentiation of gynoecium tissues. The lateral domain has been reported to reach its final thickness at stage 7 and then performs cell proliferation following the apical–basal axis in the gynoecium (Bowman et al. 1999; Roeder and Yanofsky 2006). This means that valve cells have started to differentiate, which could explain the presence of *CYCD3;2* since early stages in valves. In the same line, the vascular development requires not only cell proliferation but cell differentiation to start performing the transport (Lucas et al. 2013). The different medial tissues (transmitting tract, septum, funiculi, ovules) start to differentiate in stage 10 and form a mature gynoecium at stage 12 (Herrera-Ubaldo and de Folter 2022); this makes sense with the expression pattern of the *CYCD3;3* gene. Finally, as the *CYCD3* genes regulate cell size by endoreduplication (Sugimoto-Shirasu and Roberts 2003; Dewitte et al. 2007; Lang and Schnittger 2020), it is notable that we observed larger cells in lateral and medial tissues of the *cycd3;1–3* triple mutant. However, better resolution and extensive image analysis are required to explore this further and reach a robust conclusion. All this evidence suggests that *CYCD3;2* and *CYCD3;3* could play a role in the endocycle control and differentiation of the lateral and medial tissues in the gynoecium, but more studies are required. Note that no promoter *CYCD3;1::GUS* activity was detected in the gynoecium, but in the recently reported gynoecium expression map by Luna-García et al. (2024), *CYCD3;1* expression is detected in the medial and lateral domains of the gynoecium*.* This means that we must reserve strong conclusions on the lack of *CYCD3;1* involvement during gynoecium development.

### D3-type cyclins function is regulated by cytokinin

The D3-type cyclins have previously been documented as being induced by endogenous signals, with cytokinin being one of them (Meijer and Murray 2000; Dewitte and Murray 2003). Indeed, our study revealed that all three *CYCD3* genes are regulated by cytokinin in inflorescence tissue (Fig. 2). However, the absence of *CYCD3;1* promoter GUS activity in the gynoecium suggests that this particular *CYCD3* gene might be under cytokinin regulation in other organs, as the meristem where it has been observed before (Dewitte et al. 2007). Cytokinin effects on the gynoecium include apical–basal defects and the promotion of cell proliferation from the replum (Marsch-Martínez et al. 2012; Zúñiga-Mayo et al. 2014; Cerbantez-Bueno et al. 2020). In our research, we indeed observed these typical effects, but we also noted additional effects within the gynoecium, specifically in the septum, transmitting tract, and ovules/funiculus (Fig. 4). Notably, these effects differed in the *cycd3;1–3* triple mutant.

Cytokinin is known to induce cell division and proliferation in aerial tissues (Perales and Reddy 2012; Schaller et al. 2014). The exogenous application of cytokinin triggers cell proliferation from the replum, leading to the formation of ectopic outgrowths; however, we observed a significant reduction in this cytokinin effect in the *cycd3;1–3* triple mutant. This reduction suggests a role for the *CYCD3* genes in cell proliferation induced by cytokinin. The *CYCD3* genes have been reported to control the G1/S transition in the cell cycle, which is pivotal for enabling cell division (Sablowski and Gutierrez 2022). Hence, cytokinin appears to regulate cell proliferation in the replum through the activation of the *CYCD3* genes. Specifically, this role might be attributed to *CYCD3;2*, as it displayed significant expression in the vasculature and the proliferating tissue following cytokinin application (Fig. S4).

In addition, the effects observed in the septum, ovule/funiculus, and transmitting tract were more pronounced and prevalent in the *cycd3;1–3* triple mutant. These effects often entailed the development of additional structures or tissues (e.g., extra septum, extra transmitting tract), indicating an imbalance in cell division and differentiation. Cytokinin has been reported to play a role in both cell division and differentiation processes (Schaller et al. 2014). However, in the absence of the *CYCD3* genes in the gynoecium, these processes appear to be affected. Importantly, the *cycd3;1–3* triple mutant did not exhibit any internal effects without ectopic cytokinin application. Therefore, the role of the *CYCD3* genes in the gynoecium appears to be influenced by endogenous r stimuli, in this case, cytokinin. Then, cytokinin seems to not only regulate cell division but also cell differentiation in gynoecium development.

### D3-type genes are regulated by cytokinin response factors

The transcription factor SPT has previously been shown to activate cytokinin responses in the medial domain during gynoecium development (Reyes-Olalde et al. 2017). The expression of *SPT* is evident throughout gynoecium development, and its mutation results in a lack of septum fusion and transmitting tract development (Alvarez and Smyth 1999; Heisler et al. 2001; Groszmann et al. 2010). Notably, the effect of ectopic cytokinin application in *spt-12* mutants is reminiscent of the *cycd3;1–3* triple mutant (Reyes-Olalde et al. 2017). In our findings, we observed different effects of SPT on the three *CYCD3* genes. While *CYCD3;1* expression was upregulated in the *SPTOE* line, it did not show any alteration in the *spt-12* mutant background. Conversely, *CYCD3;2* was downregulated in *spt-12* but did not show any effect in *SPTOE*. *CYCD3;1* could be impacted by some unknown secondary factor in *SPTOE* that is not downregulated in the *spt-12* background. In the case of *CYCD3;2*, it seems to depend on *SPT* expression, but the overexpression of *SPT* has no effect which could be due to a missing factor. Additionally, it is worth noting that *CYCD3;1* and largely *CYCD3;2* do not overlap with *SPT* in the gynoecium, and their regulatory regions lack SPT binding sites, which supports the idea of indirect regulation. On the other hand, *CYCD3;3* expression nearly perfectly aligns with *SPT* in the gynoecium and appears to depend on *SPT* expression. Indeed, the regulation of *SPT* over *CYCD3;3* directly impacted a specific region of its regulatory sequence. This suggests that *SPT* modulates the activity of *CYCD3* genes through a distinct regulation mechanism for each gene.

The *spt-12* mutant exhibits a partial recovery of its wild-type phenotype following the addition of ectopic cytokinin (Reyes-Olalde et al. 2017). This implies that cytokinin can, to some extent, compensate for the absence of SPT in mediating proper gynoecium development. In our research, we found that in the *spt* mutant background, cytokinin can restore the expression of *CYCD3;2* and *CYCD3;3*. However, *CYCD3;1* does not respond to cytokinin application in the *spt* mutant background, indicating that cytokinin regulates this gene solely through genes activated by SPT, likely happening in the shoot apical meristem (SAM). *CYCD3;2* appears to be under the control of genes that respond to cytokinin and are also activated by SPT; furthermore, the regulatory mechanism may be distinct for the *CYCD3;2* expression in the medial domain versus the lateral domain. Finally, *CYCD3;3* depends on SPT and other factors associated with the cytokinin response (Fig. 5). There is evidence suggesting that STM regulates *CYCD3* expression, but this regulation has been identified as indirect through cytokinin (Scofield et al. 2013). Additionally, SPT physically and genetically interacts with other transcription factors related to gynoecium development, such as the HECATE (HEC) transcription factors (Gremski et al. 2007; Schuster et al. 2014, 2015; Herrera-Ubaldo et al. 2023). HEC functions related to auxin and cytokinin signaling and hormonal cross talk regulation in the SAM and gynoecium have been reported (Schuster et al. 2014, 2015; Gaillochet et al. 2017), though for gynoecium development, more studies must be performed to fully understand their functions. Therefore, the mechanistic link connecting cytokinin with D3-type cyclins appears to be complex and remains incompletely understood.

In conclusion, our findings suggest that *CYCD3* genes play distinct and essential roles in the development of various gynoecium tissues. These roles are influenced by cytokinin and its associated response factors.

### Supplementary Information

Below is the link to the electronic supplementary material.

Supplementary file1 (PDF 1284 KB)

## Data Availability

The datasets generated during and/or analysed during the current study are available from the corresponding author upon reasonable request.

## Abbreviations

AbA: Aureobasidin A
CMM: Carpel margin meristem
SPT: SPATULA
