# Supplementary material for: The role of D3-type cyclins is related to cytokinin and the bHLH transcription factor SPATULA in Arabidopsis gynoecium development
Source: Planta. 2024 Jul 9;260(2):48. doi: 10.1007/s00425-024-04481-4 (PMC11233295; doi:10.1007/s00425-024-04481-4)
Supplement: Supplementary file 1 — Supplementary file1 (PDF 1284 KB) [file 425_2024_4481_MOESM1_ESM.pdf]

The role of D3-type cyclins is related to cytokinin and the bHLH transcription factor SPATULA in Arabidopsis gynoecium development

Vincent E. Cerbantez-Bueno[1#†], Joanna Serwatowska[1#‡], Carolina Rodríguez-Ramos[1], J. Erik Cruz-Valderrama[1§], and Stefan de Folter[1*]

[1] Unidad de Genómica Avanzada (UGA-Langebio), Centro de Investigación y de Estudios Avanzados del Instituto Politécnico Nacional, Irapuato 36824, México.

[#]These authors contributed equally.

[†] Present Address: Department of Botany and Plant Sciences, University of California Riverside, Riverside, CA 92521, USA.

[‡] Present Address: Departamento de Ingeniería Genética, Unidad Irapuato, Centro de Investigación y de Estudios Avanzados del Instituto Politécnico Nacional, Irapuato 36824, México.

[§] Present Address: Departamento de Biología Molecular de Plantas, Instituto de Biotecnología, Universidad Nacional Autónoma de México, Avenida Universidad 2001, Colonia Chamilpa, 62210 Cuernavaca, Morelos, México.

[*] Corresponding author: Stefan de Folter, stefan.defolter@cinvestav.mx

ORCID ID: 0000-0003-4363-7274

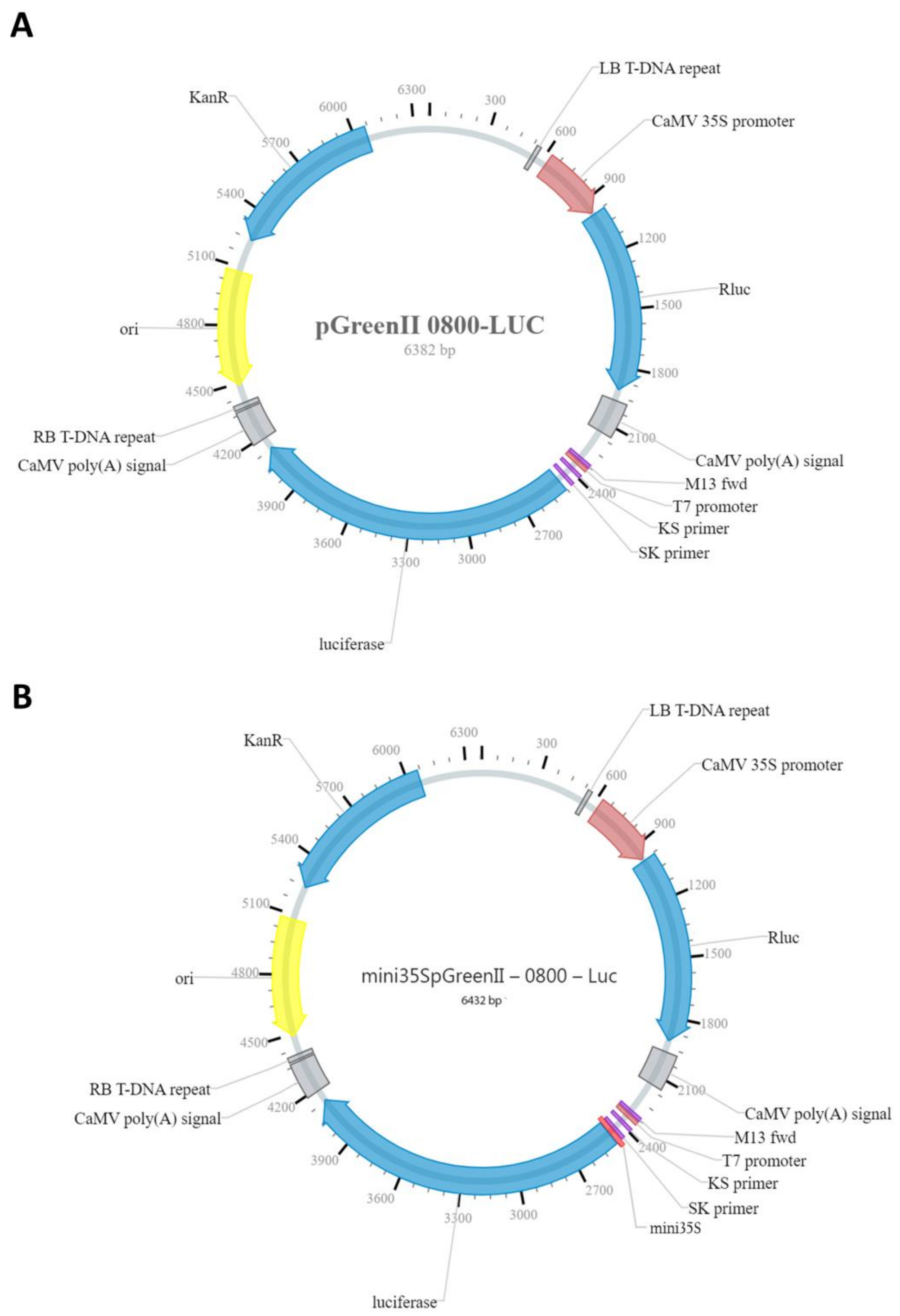

Fig. S1 pGreenII-0800-LUC and mini35S pGreenII-0800-LUC vectors.

Original version (**A**) and modified vector version (**B**).

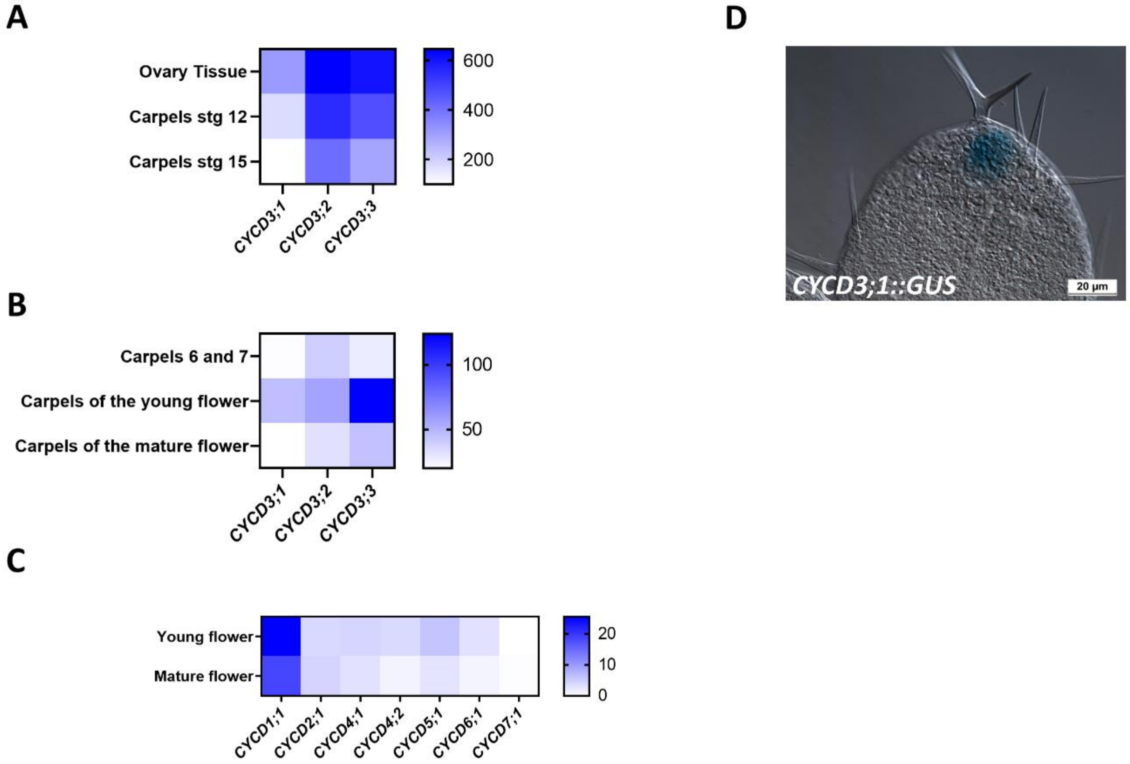

Fig. S2 Heatmaps of the database information (RNAseq and Microarrays) of the D-type *CYC* genes expression in different carpel and flower samples.

(A, B) Carpel data. **(C)** Flower tissue data. Data were obtained from Klepikova atlas and developmental map (Klepikova et al. 2016; Winter et al. 2007) in the eFP Browser. Absolute values were collected and plotted in Prisma (Graphpad). **(D)** GUS expression in a leaf tip of a *CYCD3;1::GUS* seedling. Scale bars= 20 µm **(D)**.

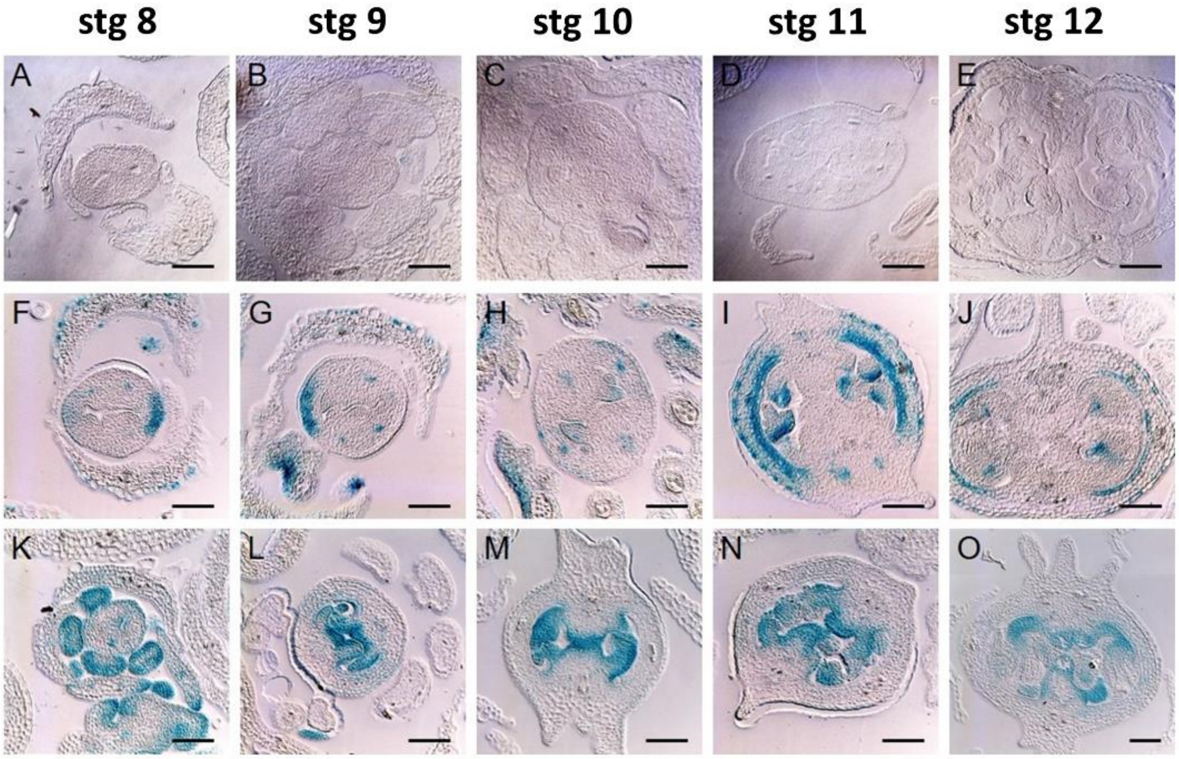

Fig. S3 Expression profile of the D3-type cyclin genes during gynoecium development after 10-day treatment with exogenous cytokinin (BAP).

(A-O) Transverse sections of the transcriptional fusion reporters *CYCD3;1::GUS* **(A-E)**, *CYCD3;2::GUS* **(F-J)**, and *CYCD3;3::GUS* **(K-O)** in stages 8 to 12 (left to right) of gynoecium development after 10-day treatment with BAP. The position of the transverse cuts is represented by arrowheads in Fig. 1s. Scale bars= 50 µm.

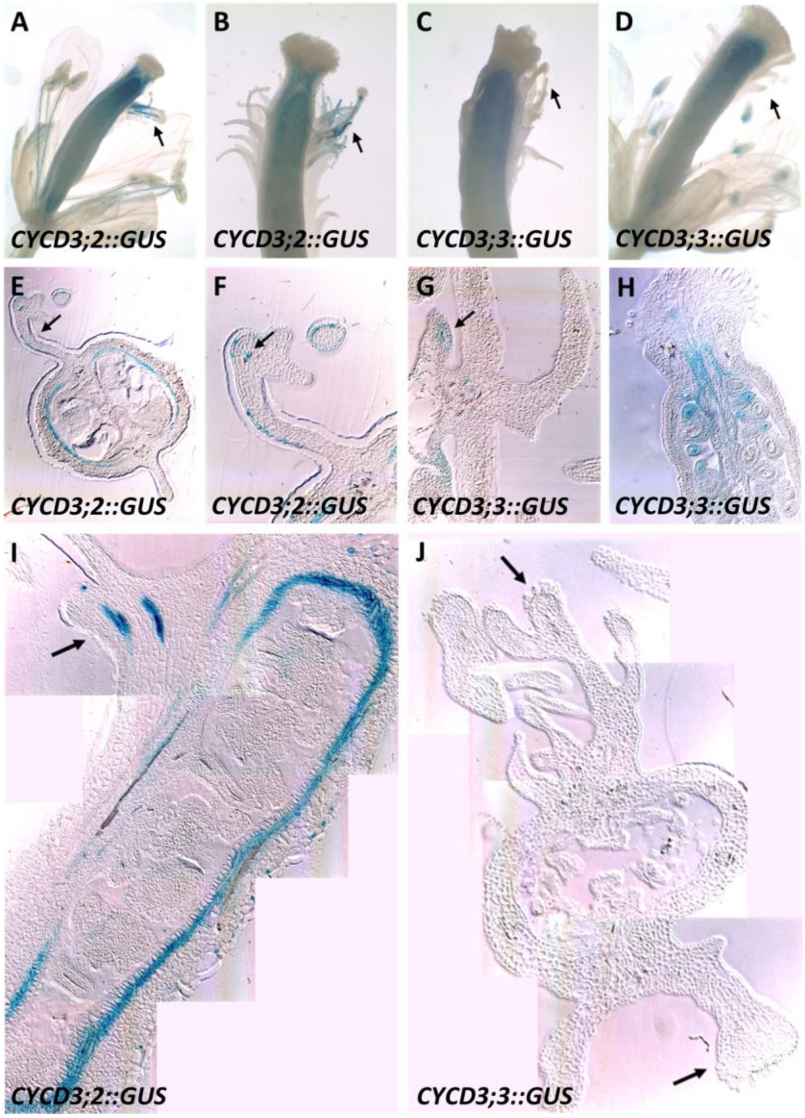

Fig. S4 *CYCD3;2* and *CYCD3;3* expression in extra vascular bundles and ectopic outgrowths after 10-day cytokinin treatment.

(A-D) Mature gynoecia of the transcriptional fusion reporter *CYCD3;2::GUS* **(A, B)** and *CYCD3;3::GUS* **(C, D)**. **(E, F)** Sections of mature gynoecia of the transcriptional fusion reporter *CYCD3;2::GUS* in transverse **(e)** and a close-up to the crest **(F)**. **(G, H)** Sections of mature gynoecia of the fusion reporter *CYCD3;3::GUS* in close up of growth crests **(G)** and stage 12 longitudinal section **(H)**. **(I, J)** Tiles of different sections of mature gynoecia of *CYCD3;2* in longitudinal **(I)** and *CYCD3;3* in transverse **()**. Scale bars= 250 µm **(A-D**; 50 µm **(E-J)**.

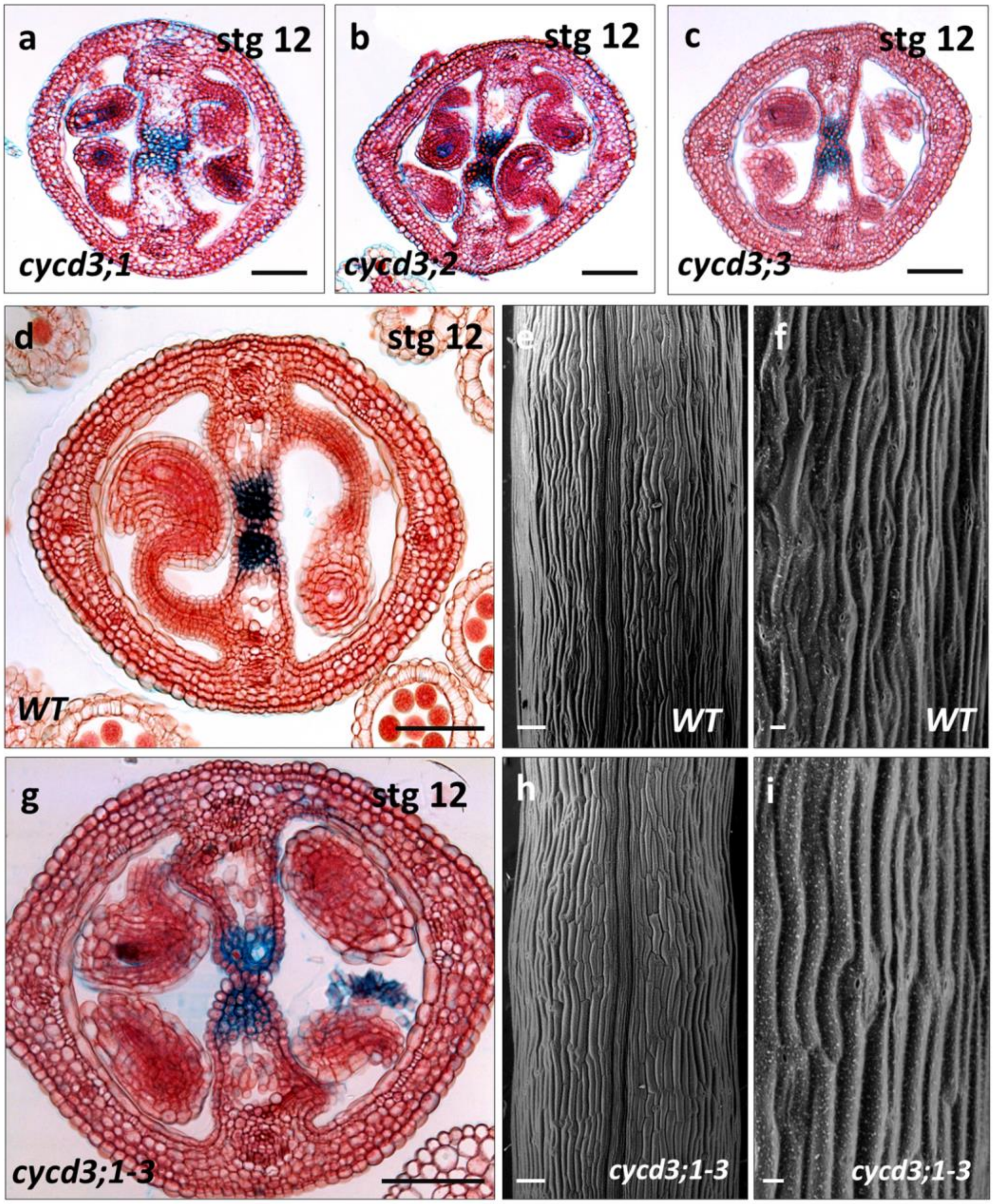

Fig. S5 Phenotypes of the single and triple D3-type cyclins mutants compared to WT.

(a-d-g) Transverse sections of the *cycd3;1* **(a)**, *cycd3;2* **(b)**, *cycd3;3* **(c)** single and *cycd3;1-3* triple **(g)** mutants compared to WT **(d)**. **(e, f, h, i)** Micrographics of the physical appearance of the epidermis in the *cycd3;1-3* **(h, j)** triple mutant compared to WT **(e, f)**. Scale bars = 50 μm **(a-d, g)**; 100 μm **(e, h)**; 20 μm **(f, i)**.

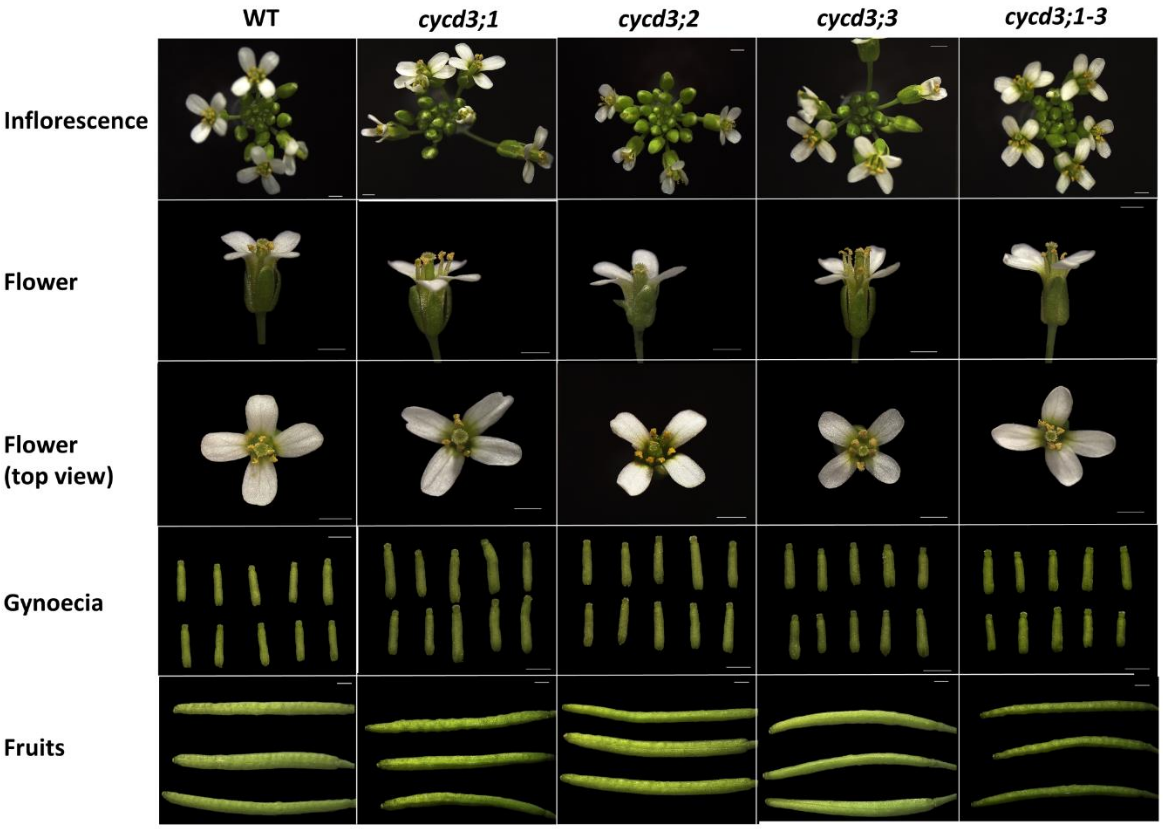

Fig. S6 Comparison of phenotypes of the single and triple mutants to WT.

Photos are from inflorescences, flowers, mature gynoecia, and fruits. Scale bars = 1 mm.

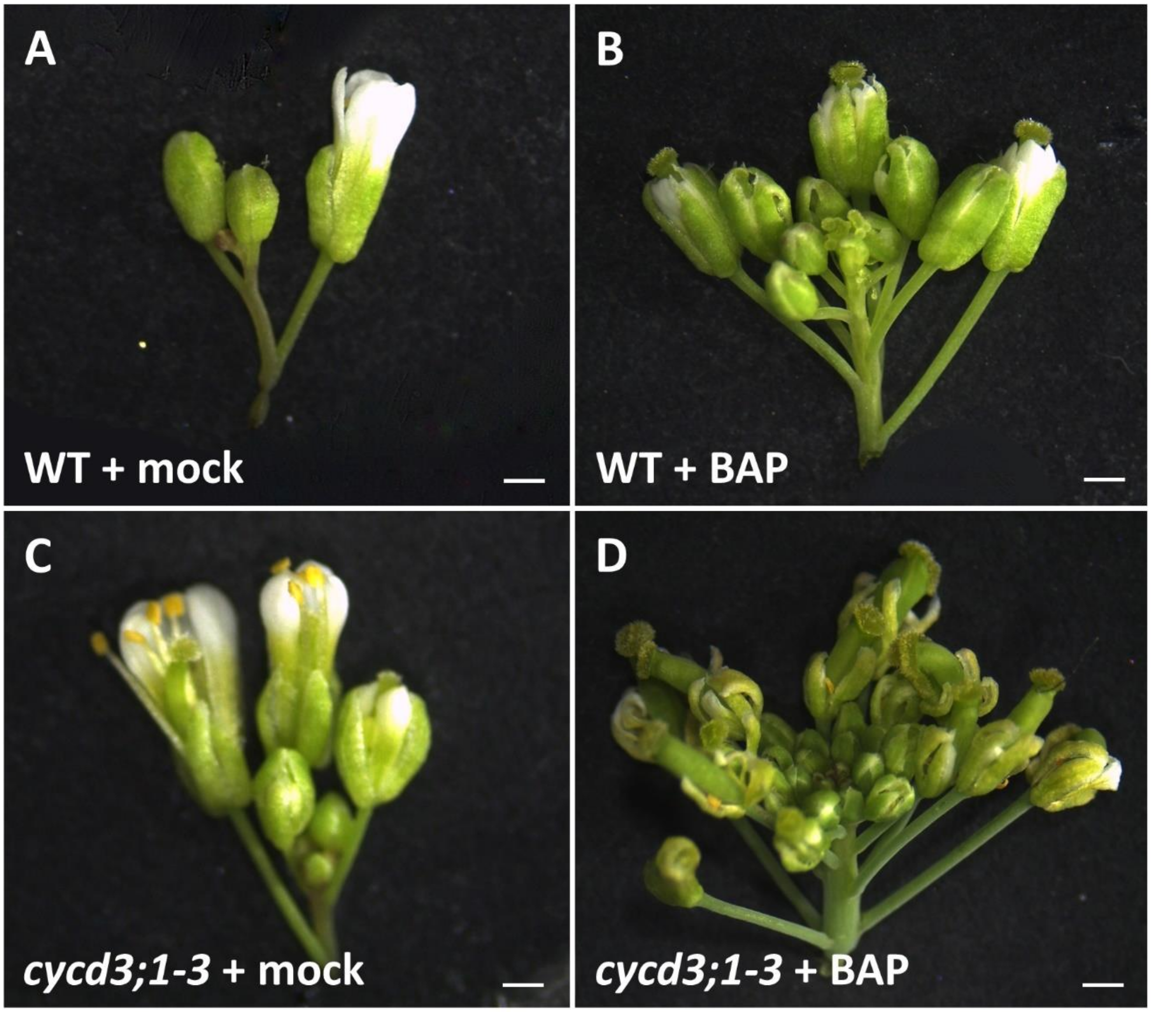

Fig. S7 Phenotypes of inflorescences of the *cycd3;1-3* mutant and WT after BAP treatment.

(A, B) Inflorescence phenotypes of WT after 10-day BAP **(B)** or mock treatment **(A)**. **(C, D)** *cycd3;1-3* triple mutant phenotypes after 10-day BAP **(D)** or mock treatment **(C)**. Scale bars = 1 mm.

Table S1. Oligonucleotide sequences used in this study.

OLIGO	F/R	SEQUENCE	INFORMATION
CYCD3;1	F	AAGCTGTTGGTTGGATTCTG	For qRT-PCR
CYCD3;1	R	GTCTCTCTGTAAGCTGTAGCT	For qRT-PCR
CYCD3;2	F	GATGACGATGAGATTCTGAG	For qRT-PCR
CYCD3;2	R	CAATCTAAAGCCTCTTTCCT	For qRT-PCR
CYCD3;3	F	ACTGCTTTGGCTTGTCTGTC	For qRT-PCR
CYCD3;3	R	GCTCCATTCTCTGTATAGTC	For qRT-PCR
CYCD3;3 I	F	CACCAAGTGAATGTATTAGAG	To amplify fragments of CYCD3;3 promoter
CYCD3;3 I	R	CGTTCCTATCGACCTAGAC	To amplify fragments of CYCD3;3 promoter
CYCD3;3 II	F	CACCACACATTGCTTTCTTTC	To amplify fragments of CYCD3;3 promoter
CYCD3;3 II	R	CTTTGTTCATTCTGCCATTAG	To amplify fragments of CYCD3;3 promoter
mini35	F	GATCCGCAAGACCCTTCCTCTATATAAGGAAGTTCATTTCATTTGGAGAGGC	To generate 35S minimum promoter
mini35	R	GGCCGCCTCTCCAAATGAAATGAACTTCCTTATATAGAGGAAGGGTCTTGCG	To generate 35S minimum promoter
